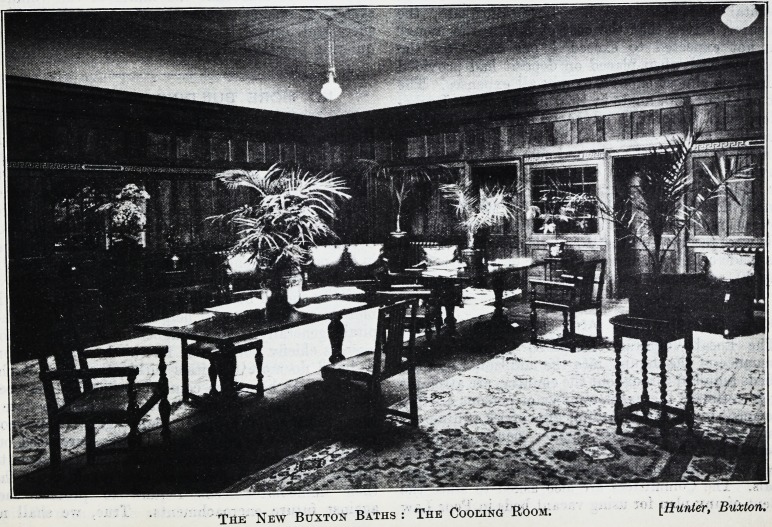# The Healing Waters of Buxton

**Published:** 1924-07

**Authors:** 


### BUXTON'S HEALING WATERS.

The new Natural Baths, which were opened at Buxton recently, are situated in the centre of the town, adjoining the south end of the famous Crescent. The front elevation is entirely in harmony with its surroundings; the entrance hall opens into a fine lounge, panelled in oak; adjoining are large and lofty bathing pools, two for men and one for women, and around them are nests of baths for individual cases, fitted with every appliance for ensuring the special treatment and comfort of the patients. The waters of Buxton have been famous since the time of the Roman occupation. Mary Queen of Scots was one of their famous patrons, and if the past of Buxton is great the Corporation have shown their faith in its future by the expenditure of £20,000 on the reconstruction of these very fine baths. Buxton enjoys enormous natural advantages in regard to scenery, health-giving associations and social attractions, and it is fitting that it should now possess one of the most modern and well-equipped bathing establishments in the country.

[Hunter, Buxton.

THE NEW BUXTON BATHS: EXTERIOR.

Nor are the poor or those of limited means neglected. For these there exists the excellent Devonshire Hospital, which has just issued its 113th Annual Report. It possesses 300 beds, and a special research organisation, complete with laboratories, has been established, charged with the duty of investigation, not only of the chemical composition of the waters, but of their physiological action.

During 1923 the number of out-patients was 376, of whom 311 were discharged as cured or much improved. The cases which most urgently need attention are those, generally, of young people, which are making rapid progress and threaten, if left to themselves, to go on to total crippling of the patient. These are given preference over other and more chronic cases, so that the disease may be arrested before permanent damage is done. They are, for the most part, obviously of infective origin, and it is worth while to give them unlimited time and attention to find out the infecting organisms.

THE NEW BUXTON BATHS: THE COOLING ROOM. [Hunter, Buxton.

## Figures and Tables

**Figure f1:**
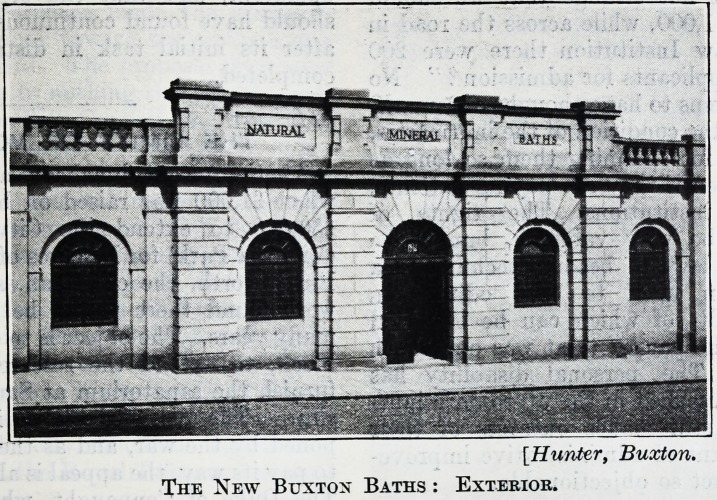


**Figure f2:**